# Experiences and Attitudes of Elementary School Students and Their Parents Toward Online Learning in China During the COVID-19 Pandemic: Questionnaire Study

**DOI:** 10.2196/24496

**Published:** 2021-05-19

**Authors:** Shu Cui, Chao Zhang, Shijiang Wang, Xingong Zhang, Lei Wang, Ling Zhang, Qiuyu Yuan, Cui Huang, Fangshuo Cheng, Kai Zhang, Xiaoqin Zhou

**Affiliations:** 1 Department of Psychiatry Chaohu Hospital Anhui Medical University Chaohu, Hefei China; 2 School of Mental Health and Psychological Sciences Anhui Medical University Hefei China; 3 Department of Pediatrics Fuyang People’s Hospital Fuyang China; 4 Department of Psychiatry The Third People’s Hospital of Fuyang Fuyang China; 5 Department of Psychiatry The Fourth Affiliated Hospital Zhejiang University School of Medicine Yiwu China

**Keywords:** attitude, elementary school students, parents, online learning, COVID-19

## Abstract

**Background:**

Due to widespread SARS-CoV-2 infection, an emergency homeschooling plan was rigorously implemented throughout China.

**Objective:**

This study aimed to investigate the experiences and attitudes of elementary school students and their parents (two generations from the same family) toward online learning in China during the pandemic.

**Methods:**

A 16-item questionnaire was distributed at the 10-day and 40-day marks after the start of the first online course to 867 parent-child pairs and 141 parent-child pairs, respectively. The questionnaire was comprised of questions pertaining to course and homework completeness, effectiveness, reliability, and abundance as well as the students’ enthusiasm for taking part in online classes and their satisfaction with the courses.

**Results:**

Our findings indicate that 90.7% (786/867) of students exhibited high or moderate enthusiasm for participating in online classes. However, most students performed poorly in online learning classes and after-school homework. With regard to satisfaction, parents' and students' average scores were 7.35 and 7.25, respectively (10-point scoring system). During the second stage of this study, parents' positive evaluations for online learning declined, including those for the effectiveness and reliability of the courses. Furthermore, the proportion of students who completed the courses and homework on time decreased; this difference proved statistically significant (*P*=.047). The parents’ and students’ overall satisfaction with online learning also declined during the second stage (parents: 7.21; students: 7.23); however, the difference in overall satisfaction between the two stages was not statistically significant (parents: *P*=.53; students: *P*=.60). Several of the parents (315/867, 36.2%) indicated that assisting with and supervising the students’ online learning resulted in increased stress. Further, 36% of parents expressed dissatisfaction with or provided suggestions for online learning; most parents and students hoped to return to face-to-face classes (parents: 823/867, 94.9%; students: 811/867, 93.5%). Finally, our results presented the following six main issues that parents were the most concerned about: (1) disappointment regarding timely interaction in courses; (2) apprehensiveness about students’ understanding of the course; (3) the increased burden of annoying adult responsibilities; (4) concern about children's eyesight; (5) the idea that teachers’ explanations were not detailed enough; and (6) concerns about the decline of students' interest in and attention toward online courses.

**Conclusions:**

Online learning can prevent the spread of infectious diseases while still allowing elementary school students to attain knowledge. However, in our study, children’s completion of the courses and homework were not satisfactory. Furthermore, their parents often experienced stress and had many concerns and complaints. Measures such as increasing the interactivity of the courses and prohibiting teachers from assigning tasks to parents could improve the effectiveness of these courses and the mental health of parents and students.

## Introduction

Due to widespread SARS-CoV-2 infection, the Chinese government postponed the opening of schools after the Spring Festival to prevent further infections [1]. Face-to-face socializing was also prohibited. China’s Ministry of Education estimated that more than 270 million students were confined to their homes, including 17.67 million elementary school students [2].

The Ministry of Education stipulated that even though schools were closed, teaching must continue during the lockdown period [3]. Accordingly, online teaching has been rigorously implemented in China [4]. Since mid-February 2020, schools and teachers of all levels have made considerable efforts toward creating and delivering online courses via internet-based methods or television broadcasts [5]. Consequently, this has resulted in the largest online learning campaign in human history.

Previous research has shown that online education has great potential for addressing the availability and efficiency of education [6,7]. However, by itself, online education is not more effective than a classroom-based approach, and its effectiveness depends on how well instructional designs are integrated into effective learning principles [8]. Differences in content quality, interactivity, and platform availability may affect learning satisfaction [9,10], but it is not clear which aspects are the most important for online education in primary schools in China. Previous studies on problems related to online learning have focused primarily on college students [11-14]. However, few studies have focused on elementary school students’ experiences and satisfaction with online learning. Another limitation that has been mentioned in previous studies is that researchers only assess online learning satisfaction from students’ perspectives. However, parents’ opinions also influence students’ satisfaction with learning and can inspire students to learn [10]. This is worrisome, as several factors (concentration, self-discipline, and related factors) can result in a host of problems during online education [15,16].

This study aimed to investigate the experiences and attitudes of Chinese elementary school students and their parents toward online learning during the COVID-19 pandemic. Furthermore, this study evaluated differences in parents’ satisfaction with online education between the 10-day and 40-day marks after the start of the first online course.

## Methods

### Ethical Approval

The study protocol was approved by the Research Ethics Committee of Chaohu Hospital of Anhui Medical University (approval number: 202001-kyxm-07). The enrolled participants received detailed explanations about this study and signed electronic informed consent forms (parents’ consent for students’ participation). All participants’ personal information was kept confidential, including their names and internet protocol addresses.

### Survey Questionnaire

The survey questionnaire was designed to assess the online learning experiences and attitudes of Chinese elementary school students and their parents (Multimedia Appendix 1) based on the concerns of parents of elementary school students and previous questionnaires [17]. Each questionnaire was completed by 1 student and 1 parent. In order to prevent selection bias from affecting the study outcomes, each parent was selected at random and chosen by their family. The questionnaire consisted of 16 items and focused primarily on obtaining basic information, including children’s grades and the equipment that was used during online classes. Thereafter, a broader selection of information was gathered, including participants’ levels of enthusiasm for online learning, the completion of online classes, the completion of assigned homework, the pressure on parents, and related factors. Items 1-12 were answered by parents and items 13-15 were answered by elementary school students. Item 16 was an open comment that was directed at parents; it was designed to obtain their opinions on online learning. To measure satisfaction, we used a 10-point scoring system (ranged from 1 to 10 with intervals of 1); 1 represented the lowest degree of satisfaction, and 10 represented the highest degree of satisfaction. The questionnaire was written in Chinese and was not translated into any other language. Primary education in China is compulsory for all children who reach a certain age (6-7 years). Primary school students are usually between the ages of 6 and 13 and are in grades 1-6. With regard to our questionnaire, primary school students only had to answer three simple questions, which they understood and correctly answered.

We pretested the questionnaire with 5 parent-child pairs of primary school students (not part of the research team) and 7 psychologists. They pretested the questionnaire to determine the feasibility and understanding of the questions and words and to provide feedback. The content validity of the final version of the questionnaire was 0.86. The Cronbach α of the questionnaire was .73, which was within the appropriate, acceptable Cronbach α range (.70-.95) [18,19]. On the basis of the Kendall sample size calculation method [20], the minimum sample size had to be 10 times the number of items in the questionnaire plus 20% of the number of invalid questionnaires. Therefore, since the scale was composed of 16 items, the minimum sample size of this study had to be 192. Our study obtained 1008 valid questionnaires and therefore met the sample size requirements.

### Survey Protocol

The questionnaire was produced and distributed by the authors. The relevant data were subsequently collected with the web-based survey tool Questionnaire Star (Ranxing Information Technology Company, Limited), a professional, web-based survey evaluation platform [21]. Questionnaire Star can be used to design questionnaires, collect data, create custom reports, and analyze results. We sent a questionnaire link to potential participants via WeChat (TenCent Holdings Limited), which is the most widely used social media platform in China.

Eligible participants included any Chinese elementary school students who participated in online education during the COVID-19 pandemic and their parents. The questionnaire survey was conducted during two separate phases in this study. A 16-item questionnaire was distributed at the 10-day and 40-day mark after the first online course. In the first phase, the questionnaire was sent to 867 parent-child pairs (867 elementary students and their parents). In the second phase, the questionnaire was sent to 141 parent-child pairs.

### Statistical Analysis

Participants’ responses were proportionally expressed and recorded with a Likert scale that was divided into “good,” “average,” and “poor” responses or “yes” and “no” responses. Continuous variables (ie, satisfaction scores) were compared with the Student *t* test. Categorical variables were compared with either chi-square tests or Fisher exact tests. Data were analyzed using PASW (Predictive Analytics SoftWare) Statistics 20 (IBM Corporation). *P* values of <.05 were considered statistically significant.

### Data Availability

All the data that support our findings are presented in the manuscript. The data sets used and analyzed during this study can be made available from the corresponding author upon reasonable request.

## Results

### Factors Affecting Students’ and Parents’ Perceptions of Online Learning

The total number of participants included 867 parent-child pairs (1 parent for each child)—867 elementary school children and 867 respective parents—during the first stage of this study. During the second stage (30 days after the first interview), only 141 parent-child pairs (1 parent for each child) were included in this study. The majority of students (304/867, 35.1%) were grade 4 students (Table 1).

With regard to the equipment used in online classes, lower-grade students were more likely to choose a television. However, grade 6 students’ following equipment choices exhibited relatively similar ratios: mobile phone (8/31, 25.8%), tablet (7/31, 22.6%), PC (8/31, 25.8%), and television (8/31, 25.8%; Table 1).

Table 1 shows the enthusiasm of primary school students in grades 1-6 who participated in online learning courses. The results revealed that most students (24/31, 37.4%) had enthusiasm for engaging in online learning courses. Surprisingly, 22.6% (7/31) of grade 6 students were not enthusiastic about taking online learning courses. This was statistically significant when compared to the enthusiasm of students in other grades (*P*=.006; Table 1).

With regard to completeness, two subitems were developed and pertained to online learning courses and the accompanying homework. Surprisingly, many of the students did not do well in the online classes or the after-class homework (Table 1). Notably, the degree to which online classes were completed was higher in both grade 1 (23/46, 50%) and grade 6 (13/31, 41.9%) than those in other grades; the difference proved statistically significant (*P*=.047; Table 1). Grade 6 students completed the largest amount of homework, while nearly half of the overall students performed well (15/31, 48.4%) (Table 1).

In this study, we designed the following three subcategories of evaluation through which the parents of elementary school students could evaluate the quality of online courses: effectiveness, reliability, and abundance. The results show that the majority of elementary school students’ parents indicated that the reliability (649/867, 74.9%), effectiveness (334/867, 38.5%), and abundance (564/867, 65.1%) of online courses were good. However, the parents’ views were inconsistent among the different grades. As such, more than 10% of grades 1 and 6 students rated the effectiveness of online classes as poor (5/46, 10.9%; 5/31, 16.1%). Furthermore, 16.1% (5/31) of the parents of grade 6 elementary students believed that the abundance of online courses was insufficient.

**Table 1:** Factors affecting student and parental perceptions of online learning at the first stage (10-day mark).

Factor		Grade 1 (n=46)	Grade 2 (n=207)	Grade 3 (n=129)	Grade 4 (n=304)	Grade 5 (n=150)	Grade 6 (n=31)	Chi-square (*df*)	*P* value
**Equipment, n (%)**								58.44 (5)	<.001
	Mobile phone	7 (15.2)	82 (39.6)	33 (25.6)	109 (35.9)	50 (33.3)	8 (25.8)		
	Tablet	16 (34.8)	14 (6.8)	20 (15.5)	33 (10.9)	20 (13.3)	7 (22.6)		
	PC	6 (13)	14 (6.8)	8 (6.2)	20 (6.6)	13 (8.7)	8 (25.8)		
	Television	17 (37)	97 (46.9)	68 (52.7)	142 (46.7)	67 (44.7)	8 (25.8)		
**Enthusiasm, n (%)**								24.494 (5)	.006
	High	27 (58.7)	153 (73.9)	103 (79.8)	247 (81.2)	123 (82)	20 (64.5)		
	Moderate	13 (28.3)	31 (15)	16 (12.4)	31 (10.2)	18 (12)	4 (12.9)		
	Low	6 (13)	23 (11.1)	10 (7.8)	26 (8.6)	9 (6)	7 (22.6)		
**Course completeness, n (%)**								18.536 (5)	.047
	Good	8 (17.4)	18 (8.7)	11 (8.5)	26 (8.6)	10 (6.7)	5 (16.1)		
	Average	15 (32.6)	39 (18.8)	23 (17.8)	47 (15.5)	28 (18.7)	8 (25.8)		
	Poor	23 (50)	150 (72.5)	95 (73.6)	231 (76)	112 (74.7)	18 (58.1)		
**Homework completeness, n (%)**								17.113 (5)	.07
	Good	5 (10.9)	17 (8.2)	14 (10.9)	21 (6.9)	9 (6)	7 (22.6)		
	Average	7 (15.2)	36 (17.4)	19 (14.7)	51 (16.8)	34 (22.7)	8 (25.8)		
	Poor	34 (73.9)	154 (74.4)	96 (74.4)	232 (76.3)	107 (71.3)	16 (51.6)		
**Course effectiveness, n (%)**								10.381 (5)	.41
	Good	19 (41.3)	79 (38.2)	53 (41.1)	116 (38.2)	59 (39.3)	8 (25.8)		
	Average	21 (45.7)	129 (58)	66 (51.1)	166 (54.6)	81 (54)	19 (61.3)		
	Poor	6 (13)	8 (3.9)	10 (7.8)	22 (7.2)	10 (6.7)	4 (12.9)		
**Course reliability, n (%)**								23.046 (5)	.01
	Good	34 (73.9)	153 (73.9)	98 (76)	236 (77.6)	110 (73.3)	18 (58.1)		
	Average	8 (17.4)	53 (25.6)	26 (20.2)	58 (19.1)	35 (23.3)	9 (29)		
	Poor	4 (8.7)	1 (0.5)	5 (3.9)	10 (3.3)	5 (3.3)	4 (12.9)		
**Course abundance, n (%)**								25.599 (5)	.004
	Good	25 (54.3)	136 (65.7)	91 (70.5)	207 (68.1)	94 (62.7)	11 (35.5)		
	Average	17 (37)	61 (29.5)	30 (23.3)	87 (28.6)	51 (34)	15 (48.4)		
	Poor	4 (8.7)	10 (4.8)	8 (6.2)	10 (3.3)	5 (3.3)	5 (16.1)		

### Parents’ Perceived Pressure From and Satisfaction With Online Learning

This study assessed the pressures that parents had to deal with during their children’s online education. This study also measured parents’ satisfaction with online learning during the COVID-19 outbreak (Table 2).

As indicated in Table 2, the parents of lower-grade students were under higher levels of pressure than parents of higher-grade students. The parents of grade 1 students (high pressure: 21/46, 45.7%) were generally the most stressed about their children's online lessons (Table 2).

With regard to satisfaction, most of the parents (675/867, 77.9%) were satisfied with the online learning courses; they scored above 6 points on the satisfaction scale (10-point scoring system; Table 2). In accordance with their parents, most of the students (641/867, 73.9%) were satisfied with their online learning courses; they also scored above 6 points on the satisfaction scale. Grade 6 students and their parents were the least satisfied with online learning, followed by grade 1 students and their parents. Interestingly, although the difference was not statistically significant (*P*=.053), grade 6 students reported higher satisfaction scores than their parents.

The results indicated that most of the parents (823/867, 94.9%) and students (811/867, 93.5%) hoped to return to face-to-face learning in their future studies (Table 2). Interestingly, 16.1% (5/31) of grade 6 students wanted to continue attending online classes in the future.

**Table 2:** Parents’ perceived pressure from and satisfaction with online learning during the first stage (10-day mark) of this study.

Variable		Grade 1 (n=46)	Grade 2 (n=207)	Grade 3 (n=129)	Grade 4 (n=304)	Grade 5 (n=150)	Grade 6 (n=31)	Chi-square (*df*)	*P* value
**Parents’ pressure, n (%)**								23.902 (5)	.008
	Low	9 (19.6)	87 (42)	56 (43.4)	124 (40.8)	82 (54.7)	12 (38.7)		
	Average	16 (34.8)	48 (23.2)	22 (17.1)	65 (21.4)	22 (14.7)	9 (29)		
	High	21 (45.7)	72 (34.8)	51 (39.5)	115 (37.8)	46 (30.7)	10 (32.3)		
**Parents’ future choice, n (%)**								11.851 (5)	.04
	Online learning	4 (8.7)	9 (4.3)	3 (2.3)	11 (3.6)	14 (9.3)	3 (9.7)		
	School	42 (91.3)	198 (95.7%)	126 (97.7)	293 (96.4)	136 (90.7)	28 (90.3)		
Parents’ satisfaction score, mean (SD)		6.83 (2.46)	7.64 (2.02)	7.12 (2.73)	7.33 (2.46)	7.51 (2.07)	6.55 (2.58)	2.198 (5)	.053
**Students’ preferred online learning, n (%)**								3.430 (5)	.63
	Yes	25 (54.3)	92 (44.7)	67 (51.9)	153 (50.3)	78 (52)	17 (54.8)		
	No	21 (45.7)	114 (55.3)	62 (48.1)	151 (49.7)	72 (48)	14 (45.2)		
**Students’ future choice, n (%)**								16.517 (5)	.006
	Online learning	3 (6.5)	10 (4.8)	8 (6.2)	12 (3.9)	18 (12)	5 (16.1)		
	School	43 (93.5)	197 (95.2)	121 (93.8)	292 (96.1)	132 (88)	26 (83.9)		
Students’ satisfaction score, mean (SD)		6.78 (2.29)	7.47 (2.35)	7.16 (2.16)	7.23 (2.40)	7.30 (2.28)	6.61 (2.67)	1.170 (5)	.32

### The Attitudes of Elementary School Students and Their Parents During the Follow-up

This study was divided into two stages. The first stage of the investigation commenced 10 days after the online course began, while the second stage started 40 days after the course began. There were no significant differences in the elementary students’ and parents’ equipment use (*P*=.35), enthusiasm (*P*=.73), stress (*P*=.96), or satisfaction (students: *P*=.60; parents: *P*=.53) between the two phases.

With regard to completeness, fewer students completed their courses (11/141, 7.8%) and after-class homework (11/141, 7.8%) during the second stage compared to those in the first stage (completed course: 238/867, 27.5%; completed homework: 228/867, 26.3%). This difference proved statistically significant (completed course: *P*<.001; completed homework: *P*<.001; Table 3). Furthermore, the parents indicated that the quality of the online courses in the second stage (effectiveness: 130/141, 92.2%; reliability: 133/141, 94.3%) was lower than that in the first stage (effectiveness: 807/867, 93.1%; reliability: 838/867, 96.7%). The difference in the number of participants who believed that courses were reliable proved statistically significant (*P*=.01; Table 3).

Parents’ and students' satisfaction levels for the online courses decreased during the second stage (parents’ satisfaction: mean 7.21, SD 2.41; students’ satisfaction: mean 7.13, SD 2.45) when compared to those in the first stage’s survey (parents’ satisfaction: mean 7.35, SD 2.35; students’ satisfaction: mean 7.25, SD 2.43); however, the difference between the two stages was not statistically significant (parents’ satisfaction: *P*=.53; students’ satisfaction: *P*=.60; Table 3).

**Table 3:** The attitudes of elementary school students and their parents during the follow-up.

Variable			Baseline (n=867)	Follow-up (n=141)	Chi-square (*df*)	*P* value
**Equipment, n (%)**					3.272 (1)	.35
	Mobile		289 (33.3)	41 (29.1)		
	Tablet		110 (12.7)	16 (11.3)		
	PC		69 (8)	8 (5.7)		
	Television		399 (46)	76 (53.9)		
**Enthusiasm, n (%)**					0.643 (1)	.73
	High		673 (77.6)	112 (79.4)		
	Moderate		113 (13)	15 (10.6)		
	Low		81 (9.3)	14 (9.9)		
**Completion, n (%)**						
	**Course**				26.130 (1)	<.001
		Good	78 (9)	1 (0.7)		
		Average	160 (18.5)	10 (7.1)		
		Poor	629 (72.5)	130 (92.2)		
	**Homework**				23.277 (1)	<.001
		Good	73 (8.4)	2 (1.4)		
		Average	155 (17.9)	9 (6.4)		
		Poor	639 (73.7)	130 (92.2)		
**Course quality, n (%)**						
	**Effectiveness**				.358 (1)	.84
		Good	334 (38.5)	51 (36.2)		
		Average	473 (54.6)	79 (56)		
		Poor	60 (6.9)	11 (7.8)		
	**Reliability**				8.715 (1)	.01
		Good	649 (74.9)	89 (63.1)		
		Average	189 (21.8)	44 (31.2)		
		Poor	29 (3.3)	8 (5.7)		
	**Abundance**				0.731 (1)	.69
		Good	564 (65.1)	96 (68.1)		
		Average	261 (30.1)	40 (28.4)		
		Poor	42 (4.8)	5 (3.5)		
**Parent factors**						
	**Pressure, n (%)**				0.083 (1)	.96
		Low	370 (42.7)	62 (44.)		
		Average	182 (21)	29 (20.6)		
		High	315 (36.3)	50 (35.5)		
	**Future choice, n (%)**				1.339 (1)	.25
		Online learning	44 (5.1)	4 (2.8)		
		School	823 (94.9)	137 (97.2)		
	Satisfaction score, mean (SD)		7.35 (2.35)	7.21 (2.41)	0.632 (1)	.53
**Student factors**						
	**Preferred online learning, n (%)**				0.955 (1)	.33
		Yes	432 (49.8)	64 (45.4)		
		No	435 (50.2)	77 (54.6)		
	**Future choice, n (%)**				0.126 (1)	.72
		Online learning	56 (6.5)	8 (5.7)		
		School	811 (93.5)	133 (94.3)		
	Satisfaction score, mean (SD)		7.25 (2.43)	7.13 (2.45)	0.525 (1)	.60

### Parents’ Open Comments Concerning Elementary School Students’ Online Education

In the open comments, participants (parents) indicated that online classes effectively used their time and network so that classes were not suspended during the COVID-19 pandemic. In terms of deficiency, parents mentioned the following six main issues: (1) disappointment regarding timely interaction in online courses; (2) worry about students not understanding the course; (3) the increased burden of annoying adult responsibilities; (4) concern regarding children's eyesight; (5) concern that teachers’ explanations were not detailed enough; and (6) concern about the decline of students' interest and attention toward online courses. We summarize the details in Textbox 1.

**Summary of parents’ open comments. In total, 73% (736/1008) of parents answered the open questions.**

**Top question**

- In total, 18.7% (188/1008) of parents thought that the interactions during the classes were inadequate.
- These parents stated that because online educational videos were taped in advance, there was a lack of question-and-answer interactions between teachers and students.
- These parents suggested that measures should be taken to ensure that teachers are aware of children's questions so that they can respond to specific questions or correct children's mistakes.

**Second highest ranked question**

- In total, 15.2% (153/1008) of parents were concerned that children could not understand the content of online educational videos.

**Third highest ranked question**

- In total, 13.6% (137/1008) of parents complained that teachers' demands, including monitoring children's online studies, checking homework, and regularly providing feedback on students’ learning, greatly increased their workload, stress, and annoyance.
- A few parents were poorly educated and could not check their children’s homework.

**Fourth highest ranked question**

- In total, 12.4% (125/1008) of parents were worried that prolonged exposure to electronic screens would lead to reduced eyesight in their children.

**Fifth highest ranked question**

- In total, 12.1% (122/1008) of parents thought that the online class durations were too short and that the teachers’ explanations were not detailed enough.
- Only 2 parents felt that the online class durations were too long.

**Sixth highest ranked question**

- In total, 3.7% (37/1008) of parents claimed that online teaching lacks a learning and competitive atmosphere and that student’s initiative and enthusiasm were not high.

## Discussion

### Principal Findings

The COVID-19 pandemic has radically changed many aspects of our lives. Furthermore, social distancing and restrictive movement policies have markedly derailed traditional educational practices [22-24]. Consequently, there is a pressing need to innovate and implement alternative education and assessment strategies [25,26]. However, the COVID-19 pandemic has provided an opportunity for the greater implementation of digital learning in elementary education that requires students to stay at home [27]. The convenience and flexibility provided by online classes seem to contribute to these classes’ proliferation and popularity [28].

Although previous studies have asserted that learners gain slightly less knowledge in online environments [29-31], our survey results (in the study’s first phase) showed that 93.1% (807/867) of parents believed that the online courses were effective and were able to convey knowledge. Conversely, a study from Ghana found that only 40 (18.7%) of their respondents agreed that they were able to learn effectively at home, while 174 (81.3%) respondents disagreed with that statement [32]. These differences may be related to the different preparation times, study content, and equipment in online courses among different countries. There is an abundance of content for online learning courses in China, as the educational content of online courses was prepared early after the onset of the pandemic. Furthermore, the courses were designed so that students could use a variety of devices to participate, including students from families that do not have internet connections; they can still access the courses through their televisions. These measures have considerably increased the effectiveness of online learning in China. However, it is worth noting that during the second survey stage, the proportion of respondents who thought that online courses were effective decreased. This may have been due to long online lessons, which make it difficult for children to concentrate, thereby reducing their productivity. The number of participants in the second stage only consisted of about one-quarter (141/867, 16.3%) of the participants in the first stage. The reason for this may have been that parents’ enthusiasm for the web-based survey declined during the second stage.

Satisfaction is a vital factor for determining the quality of online learning [33-35], as it reflects students’ pleasure and fulfillment with the different aspects of learning services [36]. This study indicated that most parents (675/867, 77.9%) were satisfied with the online learning courses; they scored above 6 points on the satisfaction scale. In accordance with their parents, most students (641/867, 73.9%) were satisfied with the online learning courses; they also scored above 6 points on the satisfaction scale. Parents’ and students’ satisfaction with the online courses decreased during the second stage; however, this did not prove to be statistically significant (parents’ satisfaction: *P*=.53; students’ satisfaction: *P*=.60).

Grade 6 students and their parents were found to be the least satisfied with online learning, followed by grade 1 students and their parents. These participants felt that the courses were ineffective and unreliable and that the content was not abundant. Therefore, for primary school students and parents, curriculum quality was closely related to satisfaction. It is important to consider that grade 6 learners are under pressure due to the junior high school entrance examinations. This is notable because online classes only teach basic knowledge and do not allow for the conduction of extracurricular classes to improve exam scores. Grade 1 students experience cognitive pressure because of their recent transition to primary school from their carefree kindergartens. Therefore, they are more likely to develop adjustment disorders, which result in poor evaluations of a curriculum’s quality.

Other studies on satisfaction have indicated that there are certain factors that affect students’ satisfaction with online learning environments, such as their interactions and self-regulation [37,38]. Parahoo et al [39] indicated that the interactions among students, teachers, and classmates are an important dimension of students’ satisfaction with online learning. Kuo and colleagues [40] found that learner-instructor and learner-content interactions are significant positive predictors of students’ satisfaction. Another study’s findings support the idea that learner-instructor interactions contribute to students’ satisfaction [41]. In our survey’s open comment section, the most frequently reported issue was the lack of interaction during online learning. This may account for the drop in satisfaction during the second survey phase. Sun and Chen [42] noted that students’ main difficulties during online learning were staying motivated, adhering to schedules, and studying regularly. Unlike the first stage of our study, only 7.8% (11/141) of students completed their courses and finished their homework on time after a 1-day online learning class. Due to the psychological characteristics of children, few elementary students are able to consistently complete their online lessons and maintain self-discipline [43].

An advantage of our study is that we were able to assess the causes of parental anxiety related to online lessons. The factor that most frequently hindered students’ learning was a lack of interaction, as identified by parents’ open comments. Online courses are taped in advance, so there was a lack of timely, two-way interactions between students and teachers. Our results indicated that students generally received information passively and lacked active communication during their online classes. Furthermore, students often did not understand certain questions. Consequently, students may lose interest in online classes over time. Another potential problem is that long online courses may result in students becoming addicted to their computers and televisions. Furthermore, prolonged exposure to computers, mobile phones, or televisions can cause vision loss in elementary school students [44].

Our results indicated that a lack of interactivity may be the most important factor affecting Chinese primary school students' satisfaction with online courses. The online lessons in this study were recorded in advance, and the videos were played to primary school students later, which resulted in the one-way flow of teaching information. This is worrisome because clear explanations and communication for clarifying questions are especially important for distance learners. In contrast, online education in high-income countries has exhibited some improvement and enhancement. They emphasized more on interactivity and student participation and considered this factor when planning online courses. For example, Hrastinski [45] provided the following theory in his research:

If we want to enhance online learning, it needs to enhance online learners' participation and interactive experience.

Suppan and colleagues [9] used a highly interactive online learning module that was tailored to customers’ timely feedback and prevented content skipping. Their results showed that their module could enhance medical students’ asynchronous distance learning in terms of knowledge acquisition. Synchronous e-learning based on interactive live webcasting has also been verified to be effective and feasible [46]. These results are consistent with our conclusions.

The low homework completion rates and high pressure on parents found in our study suggest that online learning tasks may be beyond the capacity of students and parents and may cause parent-child conflicts and emotional problems. Our data are consistent with those of another web-based survey in China, in which parents' Self-rating Anxiety Scale results showed that the degree of anxiety was higher than normal. Additionally, 17.6% of students were suspected of experiencing emotional problems during online homeschooling [47]. Another survey showed that 73.9% of primary and secondary school students’ parents felt that their burden increased, and compared to these parents, the burden on parents of primary school students in grades 1-3 increased by a higher degree (79.3%) [48].

In this study, 13.6% (137/1008) of parents complained about having to supervise their children, check their homework, and frequently deliver feedback to the teachers. This considerably increased parents’ workload, stress, and annoyance. Moreover, several parents were unable to help their children, as they were uneducated. According to a previous study, there has been an alarming increase in child abuse and domestic violence rates in Brazil during the pandemic. This may be related to families’ financial constraints, increased parental burdens resulting from school closures, parental stress, and the difficulty of dealing with children's irritability during isolation [49]. In our survey’s open comment section, a parent wrote that when he was supervising his child, the child was undisciplined in class and perfunctory in completing his homework. The parent became particularly irritable and violent and stated that he even beat the child. The reason for such conflicts may be that parents endlessly nag their children when they are supervising their children’s studies and correcting homework. This often results in children feeling that their space for independence is greatly compressed, which gives rise to conflicts between parents and students [50].

In academic circles, it is generally believed that there are utilitarian education and teaching concepts in China. Teachers who believe that “practice makes perfect” require students to perform many exercises during and after class. Since online courses in primary schools are prerecorded and lack teacher-student interaction, teachers transfer their responsibility of correcting homework to parents, which increases conflict rates and stress. Our results thus offer a new strategy for solving parent-child conflicts and emotional problems during online homeschooling.

### Limitations

There are several limitations to this study that need to be discussed. First, the sample size was not very large. In future studies, a larger sample size should be used to validate this paper’s results. Second, this study did not compare elementary school students’ tests scores from before and after online learning. Test scores can provide a more intuitive perspective on the effects of online learning. However, due to the regulations of the Ministry of Education, we were unable to obtain the scores of the elementary school students. Third, our scale does not provide demographic data, such as age, gender, or participants’ household incomes. As such, it was impossible to compare the differences among participants’ demographic data. This is problematic because elementary school students of different ages, genders, or income levels may have different experiences and attitudes toward online learning. Lastly, we did not investigate teachers' attitudes toward online learning. These issues need to be explored in future research.

### Conclusion

To the best of our knowledge, this study is the first to evaluate experiences and attitudes toward online learning among participants of two generations in the same family during the COVID-19 pandemic. Online learning can prevent the spread of infectious diseases and allow elementary school students to gain knowledge. Most enrolled elementary school students (673/867, 77.6% at baseline; 112/141,79.4% at follow-up) were very enthusiastic about participating in online classes, and students and their parents were satisfied with these classes. Students were able to adequately complete all of their lessons and after-school homework assignments during the initial phase of online learning. However, as time progressed, the percentage of students who completed their lessons and homework on time decreased. At this later stage, students’ and parents’ satisfaction with online lessons decreased. However, some online learning tasks may be beyond the capabilities of elementary school students and parents and may cause emotional and behavioral problems. This study provides evidence for policy changes that aim to reduce the amount of pressure on parents and improve mental health levels, including those that prohibit teachers from assigning the task of checking homework to parents and increase the amount of interaction between teachers and students in online classes.

## Appendix

Multimedia Appendix 1 Survey on the online education status of primary school students and the satisfaction of parents and students.

## Abbreviations

PASW: Predictive Analytics SoftWare
