# Supplementary material for: Experiences and Attitudes of Elementary School Students and Their Parents Toward Online Learning in China During the COVID-19 Pandemic: Questionnaire Study
Source: J Med Internet Res. 2021 May 19;23(5):e24496. doi: 10.2196/24496 (PMC8136302; doi:10.2196/24496)
Supplement: Multimedia Appendix 1 [file jmir_v23i5e24496_app1.docx]

**Multimedia Appendix 1: Survey on the online education status of primary school students and the satisfaction of parents and students.**

Questions 1-12 are answered by parents, and 13-15 by pupils.

Q1. What grade is your child in?

- Grade one
- Grade two
- Grade three
- Grade four
- Grade five
- Grade six

Q2. Did your child have online learning during the epidemic?

- Yes
- No

Q3. What equipment is used for online learning?

- Mobile phone
- Paid
- PC
- TV

Q4. Did your child take the enthusiasm to participate in online learning?

- Good
- Average
- Poor

Q5. Did your child finish the whole one-day course of online learning from beginning to end?

- Good
- Average
- Poor

Q6. Did your child complete the homework assigned after online learning?

- Good
- Average
- Poor

Q7. Please rate the effectiveness of online learning for your child.

- Good
- Average
- Poor

Q8. Please rate the reliability of online learning for your child.

- Good
- Average
- Poor

Q9. Did you think the school has abundant online learning resources for students?

- Good
- Average
- Poor

Q10. Did you think managing your child's online learning always gives you a sense of tension or pressure?

- Good
- Average
- Poor

Q11. Do you want your child to continue online learning or face to face classes at school after the epidemic?

- Online learning
- School

Q12. Please use 1 to ten to rate the satisfactory of online learning for your child. (One means very dissatisfied, and ten means very satisfied).

Q13. Did you like online learning? (Pupils answer this question)

- Yes
- No

Q14. Do you want to continue online learning or go back to school? (Pupils answer this question)

- Online learning
- School

Q15. Please use 1 to ten to rate the satisfactory of online learning. (One means very dissatisfied, and ten means very satisfied). (Pupils answer this question)
